# Exploring Microbial and Biophysical Aspects of Dry Skin: In Vivo Test of a Novel Skincare Formulation

**DOI:** 10.1111/jocd.70546

**Published:** 2025-11-22

**Authors:** Camilla D'Antonio, Fabrizia Lo Bosco, Giulia Gentili, Serena Belmonte, Giulia DeMartini, Giorgia Condrò, Paola Perugini

**Affiliations:** ^1^ Scientific Department Medspa Srl Milan Italy; ^2^ Etichub s.r.l., Academic Spin‐Off University of Pavia Pavia Italy; ^3^ Department of Drug Sciences University of Pavia Pavia Italy

**Keywords:** biophysical parameters, dry skin, postbiotics, prebiotics, probiotics, skin microbiota

## Abstract

**Background:**

Dry skin is a prevalent dermatological concern influenced by environmental factors and characterized by compromised skin barrier integrity. In addition to this, skin microbiota is involved and plays a crucial role in maintaining microbial flora balance. This study aims to investigate dry skin condition and microbiota then the efficacy of a novel skincare formulation (Nourish 3‐Biotic Rich Serum) containing prebiotics, probiotics and postbiotics in addressing dry skin symptoms and rebalancing skin microbiota. They are mainly microorganism lysate, together with fermented substances such as oligosaccharides, enzymes, and peptides, that support and provide nourishment to bacteria that live on the skin.

**Materials and Methods:**

Serum formula and serum placebo were tested in a single‐blind study in which 20 female volunteers aged 28–65 with dry skin were enrolled. Several skin biophysical parameters related to skin barrier condition (*stratum corneum* water content, transepidermal water loss (TEWL), protein content), mechanical properties (*R*_0_, *R*_2_) and topographic appearance (skin roughness) and skin microbiota analysis were monitored over 30 days of daily application of the products in a split‐face protocol. To better understand the results, the selected panel was then divided into two groups based on different ages: younger (< 50 years) and older (> 50 years).

**Results and Conclusion:**

This study highlighted that the product has proven to have a moisturizing action, exerts a cellular renewal action, improves skin firmness and elasticity, and reduces skin roughness. For microbial analysis, the main evidence is that both the placebo and the product help restore the skin microbiota but only the product increased the quantity of *Cutibacterium acnes* (one of the primary commensal bacteria of the skin) and was able to decrease the abundance of *Pseudomonas koreensis* usually associated with dry and rosacea skin with beneficial effects.

## 1 Introduction

Human skin is constantly exposed to a wide range of environmental factors that affect its health and appearance. Among these effects, the increasing prevalence of dry skin has become a significant concern for many people, sometimes causing considerable discomfort and distress. The etiology of this condition is multifaceted. Air pollution, exposure to UV radiation, and abrupt climate changes are among the myriad influences that can compromise the skin barrier, rendering it susceptible to dryness and dehydration [1, 2]. Consequently, it becomes fundamental to implement appropriate precautions and a tailored skincare beauty routine to effectively counter the daily challenges. Dry skin is characterized by inadequate hydration and compromised barrier integrity, heralded by symptoms such as skin flaking, sensitivity, and tightening [3]. Predominantly affected areas include the cheeks, forehead, and nose, often accompanied by sensations of itching and discomfort [4]. Diminished elasticity exacerbates the prominence of wrinkles and fine lines, while the rough texture and heightened susceptibility to irritants and extreme weather conditions further highlight this condition. At the cellular level, dry skin is related to the disruption in the skin barrier, stemming from altered proliferation and differentiation of epidermal cells, along with imbalances in lipid synthesis and natural moisturizing factor (NMF) production. Observable, tactile, and sensory manifestations of skin dryness encompass visible alterations such as redness, flakes, and cracks, accompanied by textural roughness and discomfort, evoking sensations of stinging or tingling. Crucial to skin function, corneocytes and their intercellular structures serve as both physical and chemical barriers against external stressors. Mechanical resilience is conferred by tightly packed keratin bundles and cross‐linked cornified envelope proteins [5].

Conversely, dehydration leads to stiffening and brittleness. Additionally, corneocytes harbor a water‐soluble fraction of humectant molecules, constituting the NMF, which contributes 10%–15% of the weight of the *stratum corneum*, the skin's outermost layer made up of dead cells. When the stratum corneum is compromised, the results are cell cohesion reduction, facilitating the infiltration of irritants, allergens, and pathogenic bacteria. Enhanced transdermal water loss due to compromised cell adhesion culminates in reduced hydration levels compared to healthy skin [6].

However, dry skin is not only correlated with itching and flaking of the surface layer, but other output is related to skin microbiota imbalance. All commensal bacteria, fungi, viruses that populate not only the skin surface but also its deeper layers are included in the skin microbiota definition [7]—for example, Firmicutes, Bacteroidetes, Actinobacteria, and Proteobacteria are the most represented phyla. Skin microbiota is fundamental to manage and to defend the body from the penetration of pathogenic bacteria and therefore to avoid the emergence of skin dysbiosis. This is mainly due to bacteria that can secrete antimicrobial peptides and anti‐inflammatory cytokines. If the microbial flora is balanced, skin results healthier, also increasing skin barrier function [8, 9].

There are many works in available literature demonstrating that dry skin is marked by a prevalence of a specific bacteria, *Staphylococcus aureus* . It is a gram‐positive bacterium that commonly colonizes human skin and mucous membranes. It can cause a wide range of infections, from skin and soft tissue infections to more severe infections. It is also known for its ability to develop antibiotic resistance, making it a challenging pathogen to treat in some circumstances [10, 11, 12].

Mitigation of dry skin is achieved through various cosmetic strategies aimed at restoring hydration and fortifying the skin barrier. Moisturizing creams incorporating humectants such as hyaluronic acid, glycerin, and panthenol enhance moisture retention, while natural oils like sweet almond and coconut oil provide hydration and essential nutrients to the skin. Ceramide‐enriched products boost the protective barrier, and gentle exfoliating products facilitate cell turnover and removal of dead skin cells [13, 14].

In efforts to restore and rebalance the skin microbiota, skincare formulations leverage prebiotics, probiotics, and postbiotics. Probiotics are defined as live microorganisms that, when administered in adequate amounts, confer a health benefit on the host, while prebiotics are non‐digestible ingredients that positively influence the host by selectively stimulating the growth and the activity of one or more bacteria. A postbiotic instead, is a bioactive compound produced by beneficial bacteria during fermentation or metabolic processes. These compounds can include various metabolites, enzymes, peptides, organic acids, and cell wall components. Postbiotics are believed to contribute to the health benefits associated with probiotics, although they do not contain live microorganisms themselves [15]. They have been recognized for their beneficial effects on gastrointestinal health. The potential modulation of microbial communities extends to skin health, underscoring their application in skincare formulations. They are increasingly being studied for their potential therapeutic applications in promoting gut health, modulating the immune system, and maintaining overall well‐being [16]. Prebiotics, including oligosaccharides, inulin, and fructo‐oligosaccharides, nourish beneficial skin microbes, while probiotics such as *Lactobacillus* and *Bifidobacterium* support the skin barrier function [17]. These ingredients are commonly integrated into cleansers, moisturizers, serums, and masks tailored to address dryness, sensitivity, and inflammation, thereby fostering overall skin health. Notably, the integration of probiotics in cosmetics poses significant challenges, particularly concerning the stabilization of live bacterial populations and adherence to microbiological safety guidelines. Nevertheless, semi‐active or non‐replicating bacterial preparations offer promising alternatives. Additionally, bacterial extracts like *Bacillus coagulans* , *Lactobacillus casei* , and *Lactobacillus acidophilus* demonstrate antimicrobial properties and beneficial effects on skin health. Further exploration is warranted to elucidate mechanisms of action and optimize their utilization in cosmetic formulations [18, 19].

In cosmetic field, many products claim to be “skin microbiota friendly” but only a few in vivo tests are validated to support it. Moreover, in literature, there isn't any study regarding dry skin compared to microbial flora and biophysical parameters. The aim of this study is to test a new formulation based on prebiotics, probiotics, and postbiotics on a panel of female volunteers aged between 28 and 65 years. For this purpose, the evaluation of skin microbiota was conducted using a protocol previously established and validated in another work already published [19]. Biophysical parameters characterizing dry skin, such as trans epidermal water loss (TEWL), *stratum corneum* water content, degree of skin desquamation and skin roughness, firmness and elasticity, were assessed using probes that do not cause pain or discomfort to the individuals. For each volunteer, the final formula and its respective placebo were tested.

## 2 Materials and Methods

### 2.1 Materials

The study included two test products: an active serum and a placebo formulation, whose compositions are detailed below. Serum formula: Aqua (Water), Prunus Amygdalus Dulcis (Sweet Almond) Oil, Dicaprylyl Ether, Butylene Glycol, Hexylene Glycol, Isosorbide Dicaprylate, Cetyl Alcohol, C13‐15 Alkane, Squalane, Lactobacillus Ferment, Fructose, Glucose, Alpha‐Glucan Oligosaccharide, Polymnia Sonchifolia Root Juice, Sphingomonas Ferment Extract, Scutellaria Baicalensis Root Extract, Lactobacillus, Potassium Cetyl Phosphate, Maltodextrin, Urea, Pentaerythrityl Tetra‐Di‐T‐Butyl Hydroxyhydrocinnamate, Sucrose, Dextrin, Alanine, Glutamic Acid, Aspartic Acid, Xanthan Gum, 1,2‐Hexanediol, Caprylyl Glycol, Lactic Acid, Stearic Acid, Sodium Benzoate, Microcrystalline Cellulose, Potassium Sorbate, Trisodium Ethylenediamine Disuccinate, Cellulose Gum, Sodium Hydroxide, Sodium Glycerophosphate, Alpha‐Isomethyl Ionone, Benzyl Salicylate, Parfum (Fragrance), CI 77491 (Iron Oxide), CI 77499 (Iron Oxide).

Placebo serum: Aqua, Trisodium Ethylenediamine Disuccinate, Microcrystalline Cellulose, Cellulose Gum, Xanthan Gum, Dicaprylyl Ether, Prunus Amygdalus Dulcis Oil, Stearic Acid, Cetyl Alcohol, C13‐15 Alkane, Squalane, Potassium Cetyl Phosphate/Sodium, Stearoyl Glutamate, Pentaerythrityl Di‐T‐Butyl, Hydroxyhydrocinnamate, Butylene Glycol, CI 77491, Sodium Glycerophosphate, CI 77499, Parfum, 1,2‐Hexanediol, Caprylyl Glycol, Lactic Acid, Sodium Benzoate, Potassium Sorbate.

### 2.2 In Vivo Study Design

For the in vivo study women aged 28–65 were selected and the protocol set up in a previous work was used [19]. The inclusion criterion was the presence of facial dryness, with a preliminary screening performed to ensure that all participants met this criterion. Women with dermatological diseases were excluded. All volunteers before undergoing the study, signed a consent according to Italian law (GDPR 2016/679) and the analyses were carried out based on the Helsinki declaration [20].

For biophysical parameters, 20 women were selected, and the cheeks were selected as the test site, as they are widely recognized in clinical research as a standard area for skin measurements, supporting methodological consistency and data comparability. The study was conducted as a single‐blind trial, and volunteers were instructed to apply the product to one half of their face and the placebo to the other half twice per day. All volunteers were informed and instructed to follow a 12‐h washout period before each visit, during which no topical products were applied to the test area. The parameters were taken before the beginning of the study (T0) and after 30 days (T30 days).

As a preliminary clinical trial, microbiota analysis was optimized on a smaller subset of 15 participants to ensure feasibility and methodological refinement based on a validated protocol [19] showing that reliable correlations between skin microbiota and biophysical parameters can be obtained with a well‐selected participant sample. The samples were collected at the beginning (T0) and after 30 days (T30 days).

### 2.3 Precautions Before Analyses

Before conducting the analyses, the laboratory was ventilated, and every surface and instrument was disinfected with ethanol. Subsequently, the operators wore gowns, gloves, and masks, and took care to sanitize each workstation after analyzing each volunteer. Skin properties were assessed using non‐invasive probes that do not cause pain or discomfort. Measurements were made in a temperature and humidity‐controlled laboratory environment (*T* = 22°C, relative humidity [RH] = 70% ± 5%) after a 15‐min adaptation period at rest.

### 2.4 Biophysical Parameters

As previously indicated, several biophysical parameters were investigated to objectively characterize the state of the skin.

For the assessment of stratum corneum water content (SCWC) an arbitrary scale (0–100 a.u.) was used through a Corneometer CM 825 (Courage&Khazaka, Cologne, Germany). The device is equipped with a 49 mm^2^ surface probe that allows measurement within a 10–20 μm depth range in the stratum corneum. TEWL (0 a 90 g/m^2^h) was analyzed with a skin evaporimeter made of a small cylindrical open chamber (1 cm in diameter, 2 cm in height) with a couple of hygrometric sensors connected to a microprocessor plugged into a computer workstation (TM 300 W, Courage&Khazaka, Cologne, Germany). Both the probes are connected to the Cutometer MPA 580 base system using the software MPA CTplus.

The degree of skin desquamation was defined by surface protein content analysis. The measurement of the protein content of the stratum corneum was done using the infra‐red densitometry (IRD) technique using the SquameScan TM (Cuderm, Dallas, TX, USA). The tape‐stripping techniques were used. Tape‐stripping is one of the most frequently used approaches to sample the *stratum corneum*. This method involves the application of an adhesive tape, 14 mm diameter (D101‐ D‐Squame Stripping Discs, Cuderm, Dallas, TX, USA), to the skin and its subsequent removal to “strip” off a layer of *stratum corneum*. A constant pression of 225 g/cm^2^ is impressed on the disc surface. The results are expressed as protein content μg/cm^2^.

The viscoelastic properties of the skin were analyzed by the DermaLab Combo elasticity probe (Cortex Technology, Hadsund, Denmark) with the air pressure‐induced skin elevation and subsequent retraction time. A negative pressure of 400 mbar is applied to the skin through a 10 mm diameter probe. Each aspiration is followed by a release time allowing the skin to return to its resting conditions. The parameters are skin firmness or softness (*R*_0_) and total elasticity (*R*_2_).

Skin roughness was studied using high‐resolution 3D images of skin taken with Antera 3D (Miravex Limited, Dublin, Ireland), equipped with complex mathematical algorithms. The investigated topographic parameter is maximum height of the roughness (mm).

### 2.5 Sample Microbial Collection

The sampling procedure for flora collection followed the protocol set up in a previous study [19]. To sum up, adhesive disc tape D‐squame (Clinical and Derm, 12221 Merit Dr. Ste. 940 Dallas, TX 75251, United States) was placed on the cheeks of each person for 10 s applying a constant pressure. Then, the adhesive pad was removed from the skin and put in a sterile Eppendorf ready for the microbial flora analyses. The procedure was repeated three times for each site. The forehead was used as a control area.

### 2.6 Skin Microbiota Analyses Methods

Libraries were prepared by following the Illumina 16S Metagenomic Sequencing Library Preparation protocol in two amplification steps: an initial 30‐cycle PCR amplification using locus‐specific PCR primers and a subsequent amplification that integrates relevant flow‐cell binding domains and unique indices (NexteraXT Index Kit, FC‐131‐1001/FC‐13‐1002). Target‐specific amplification was performed by using: 16S 341F 5′‐CCTACGGGNGGCWGCAG‐3′ and 16S 805R 5′‐GACTACHVGGGTATCTAATCC‐3′. Libraries were sequenced on the MiSeq instrument (Illumina, San Diego, CA) using 300‐bp paired‐end mode.

### 2.7 Skin Microbiota Diversity

To classify groups of closely related individuals based on their genetic sequences, the observed OTUs parameter was used. It refers to the number of Operational Taxonomic Units identified in the samples.

To evaluate the diversity of facial skin microbiota between the product and placebo, alpha diversity analysis was measured. Diversity indices included the Shannon and Simpson indices, along with richness estimators such as Chao1, sequencing depth index (goods coverage). QIIME 2 software was employed for data elaboration. The Shannon Diversity Index is a commonly used metric to quantify the diversity within a community, including microbial communities like skin microbiota. It provides valuable insights into the stability and health of these microbial populations across different environments. Similarly, the Simpson Diversity Index considers both the richness and evenness of species, being particularly sensitive to changes in the abundance of dominant species within the community. The Chao1 index helps estimate the true diversity of microbial species by accounting for potential rare or undetected species within each sample. Additionally, the concept of “good coverage” indicates the extent to which sequencing effectively covers the genetic material of interest. This measure is crucial for ensuring the reliability and depth of sequencing data, as it reflects how thoroughly each base of the DNA sequence was sampled during the process.

### 2.8 Statistical Analysis

The data obtained are processed either as descriptive statistical analysis or through statistical analysis with specific comparison tests for parametric and non‐parametric data. The type of statistical analysis applied to the data depends on the distribution of the data and the specific characteristics of the sample. To perform an accurate comparison between different groups or between measurement times (T1 and T0), appropriate statistical tests are used. The choice of test depends on the nature of the data and the type of comparison desired.

- Intra‐group comparison (between Ti and T0 values): Intra‐group comparison is used to assess differences within the same group of subjects by comparing data collected at time Ti with data collected at time T0. For this comparison, we use Student's *t*‐test for paired data, which is appropriate when we want to check whether the mean of the values changes significantly between the two time points.
- Intergroup comparison (between groups T0 or between groups Ti): In this case, average values are compared between different groups, for example between values at time T0 of distinct groups or between values at time Ti. If the data follow a normal distribution, the intergroup comparison is performed using Student's *t*‐test for independent samples. If the data distribution is not normal or more than two groups are compared, the ANOVA (Analysis of Variance) test is used to determine whether there are statistically significant differences between the means of three or more groups.

The software used to perform the statistical analysis is the GraphPad Prism, a tool for managing, analyzing and visualizing data using advanced statistical techniques.

A significance level of 5% was chosen; therefore, variances are considered statistically significant at *p* < 0.05.

## 3 Results and Discussion

### 3.1 Results

Generally, skin is classified into three categories: dry, combination and normal. Additionally, sensitive skin can be a characteristic of any of these three categories. Dry skin is characterized by flakiness, and increased irritation, especially in the nasolabial, cheek, and forehead areas [3, 21]. This phenomenon is due to a decreased integrity of the skin barrier, leading corneocytes to be less adherent to each other, resulting in increased transepidermal water loss exacerbating the condition. Another significant factor is an imbalance in skin microbiota. This occurs because the compromised skin barrier allows penetration of allergens, irritants, and pathogenic bacteria, leading to inflammation and irritation. Hence, among the various substances used in cosmetic applications, pre‐, post‐, and probiotics play a fundamental role in restoring the skin's bacterial flora. The aim of this study was to test the efficacy of a new cosmetic serum (Miamo, Nourish 3‐Biotic Rich Serum) intended for dry skin with altered microbiota. To this end, 20 female volunteers were recruited for the analysis of biophysical parameters, and the first 15 were additionally selected for microbiota sampling on the cheeks. The product was compared with a placebo for proper analysis and data accuracy. The study lasted 30 days and the study was a single blind not to influence volunteers.

### 3.2 Biophysical Parameters

To test the efficacy of the new formula, different biophysical parameters were taken before the application of the products (T0), after 30 days (T30 days). The selected panel was divided into two groups based on different ages: younger (< 50 y.o), and older (> 50 y.o.). The results are presented below divided per skin barrier parameters (*stratum corneum* water content, TEWL, and protein content) and topographic and viscoelastic parameters (roughness, *R*_0_, and *R*_2_).

#### 3.2.1 Skin Barrier Parameters

Skin barrier parameters, such as stratum corneum water content, transepidermal water loss (TEWL), and protein content, are essential indicators of the skin's functional integrity and its ability to maintain moisture and overall functional cohesion.

##### 3.2.1.1 Stratum Corneum Water Content (SCWC)

Table 1 reports the results of the SCWC expressed as mean ± standard deviation of product and placebo at different times for the entire panel as well as the age‐specific subgroups.

**TABLE 1:** SCWC results on product and placebo.

	Total		Yg		Og	
	Mean	SD	Mean	SD	Mean	SD
Product						
T0	34.90	3.47	34.89	2.41	34.90	4.43
T30 days	40.77	4.08	41.03	2.48	40.50	5.37
Var%	16.94	**—**	17.76	**—**	16.12	**—**
*p*‐value	< 0.0001****	**—**	< 0.0001****	**—**	< 0.0001****	**—**
Placebo						
T0	35.84	4.59	36.07	3.55	35.62	5.64
T30 days	35.43	4.37	35.24	3.64	35.63	5.19
Var%	−0.95	**—**	−2.21	**—**	0.31	**—**
*p*‐value	0.3162	**—**	0.2033	**—**	0.9895	**—**

*Note:* Statistically significant as *p*‐value < 0.05.

**** *p* ≤ 0.0001 extremely statistically significant.

The instrumental analysis at T0 confirmed that the subjects indeed had dry skin as per the inclusion criteria, with the recorded values being highly representative of this skin type.

The difference at T0 between product and placebo is not significantly different (*p* = 0.1321), showing that the test was conducted correctly. The parameter investigated globally increases by 16.94% after 30 days of product application (statistically significant).

When analyzing the results by age, the investigated parameter shows a comparable increase in both groups (Yg: 17.76%; Og: 16.12%), indicating an action across all the demographic range. Therefore, the product can be considered effective in improving dry skin condition uniformly in both younger and older groups. Statistical analysis comparing younger and older groups at baseline (T0) and after 30 days of treatment (T30) revealed no significant differences (*p* = 0.6842 and *p* = 0.7544, respectively), supporting the observation that the product exerts a uniform effect across age groups.

##### 3.2.1.2 Trans Epidermal Water Loss (TEWL)

Table 2 shows all the results of TEWL for the entire panel as well as the age‐specific subgroups.

**TABLE 2:** TEWL results on product and placebo.

	Total		Yg		Og	
	Mean	SD	Mean	SD	Mean	SD
Product						
T0	11.03	1.06	11.25	0.60	10.80	1.38
T30 days	10.85	1.29	11.00	1.13	10.69	1.47
Var%	−1.46	—	−2.34	—	−0.57	—
*p*‐value	0.4390	—	0.3575	—	0.7889	—
Placebo						
T0	11.14	1.10	10.96	0.67	11.32	1.44
T30 days	11.37	1.31	11.29	1.32	11.45	1.37
Var%	2.41	—	2.83	—	2.00	—
*p*‐value	0.3937	—	0.2722	—	0.7766	—

*Note:* Statistically significant as *p*‐value < 0.05.

The investigated parameter demonstrates that, despite being dry, the skin generally does not show alterations and that the skin remains intact over time. In all cases, the values stay within the physiological range after 30 days of product application with no significant changes. Furthermore, statistical analysis comparing younger and older groups revealed no significant differences at baseline (T0, *p* = 0.2101) or after treatment (T30, *p* = 0.4935), indicating that the parameter is consistently maintained across age groups.

##### 3.2.1.3 Protein Content of the Stratum Corneum

The amount of protein in the stratum corneum is an index of desquamation or cohesion of the skin barrier and this parameter can be used to assess the efficacy of the product used to exfoliate the surface layer of the stratum corneum to promote the renewal of the skin. Table 3 reports the results of the product and placebo at different times for the entire panel as well as the age‐specific subgroups.

**TABLE 3:** Protein content results on product and placebo.

	Total		Yg		Og	
	Mean	SD	Mean	SD	Mean	SD
Product						
T0	15.09	3.60	14.55	4.18	15.63	3.03
T30 days	12.42	2.00	12.82	2.26	12.03	1.74
Var%	−14.57	—	−8.41	—	−20.74	—
*p*‐value	0.0037**	—	0.1696	—	0.0096**	—
Placebo						
T0	14.30	3.23	13.67	3.57	14.92	2.89
T30 days	14.40	2.48	14.38	2.71	14.43	2.37
Var%	3.63	—	8.13	—	**−0.87**	—
*p*‐value	0.8671	—	0.3895	—	0.6374	—

*Note:* Statistically significant as *p*‐value < 0.05.

** *p* ≤ 0.01 statistically significant.

**FIGURE 1:**
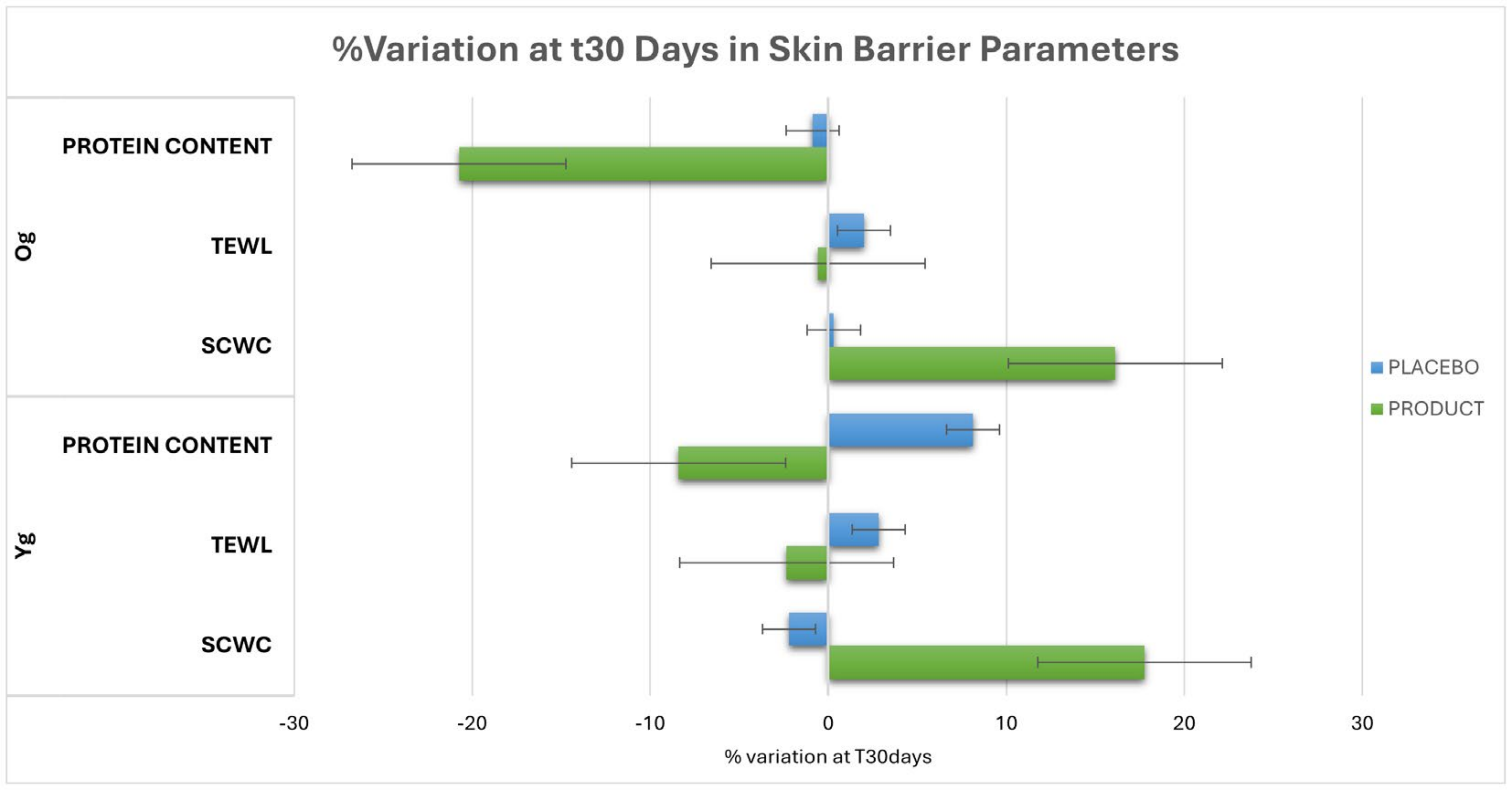
Summary of skin barrier parameter variations between the two age groups (Yg; Og). The image illustrates differences in hydration, transepidermal water loss (TEWL), and protein content, highlighting the outcomes across different age demographics after 30 days of product application.

The difference at T0 between product and placebo is not significantly different (*p* = 0.2889), showing that the test was conducted correctly. The investigated parameter, indicative of the stratum corneum protein content, decreases by −14.57% after 30 days of product application (statistically significant) demonstrating the ability to improve the corneocyte cohesion. In this case, the age subdivision reveals different outcomes: the parameter decreases significantly for the older group (−20.74%), where the combination of dry skin and age‐related factors likely contributes to a greater efficacy of the product, resulting in enhanced cohesion of the corneocytes after 30 days of application. In contrast, the reduction of 8.41% is not significant for the younger group (Figure 1).

#### 3.2.2 Topographic and Visco‐Elastic Parameters

Topographic and viscoelastic parameters, such as roughness, *R*_0_, and *R*_2_, are critical for assessing skin surface characteristics and mechanical properties. These parameters describe skin's texture and elasticity, which are essential for evaluating the performance of skincare treatments.

##### 3.2.2.1 Elasticity

Tables 4 and 5 report the results of elasticity parameters (*R*_0_ and *R*_2_) as mean ± standard deviation of product and placebo at different times for the entire panel as well as the age‐specific subgroups.

**TABLE 4:** *R*_0_ results on product and placebo.

	Total		Yg		Og	
	Mean	SD	Mean	SD	Mean	SD
Product						
T0	2.849	0.427	2.879	0.348	2.820	0.511
T30 days	2.667	0.303	2.720	0.266	2.614	0.342
Var%	−5.76	—	−5.18	—	−6.33	—
*p*‐value	0.0004***	—	0.0046**	—	0.0225*	—
Placebo						
T0	2.822	0.414	2.912	0.409	2.731	0.420
T30 days	2.866	0.373	2.927	0.366	2.804	0.389
Var%	1.90	—	0.75	—	3.04	—
*p*‐value	0.0565	—	0.6001	—	0.0492*	—

*Note:* Statistically significant as *p*‐value < 0.05.

* *p* ≤ 0.05 low statistically significant.

** *p* ≤ 0.01 statistically significant.

*** *p* ≤ 0.001 highly statistically significant.

**TABLE 5:** *R*_2_ results on product and placebo.

	Total		Yg		Og	
	Mean	SD	Mean	SD	Mean	SD
Product						
T0	0.860	0.041	0.874	0.036	0.846	0.042
T30 days	0.931	0.048	0.943	0.050	0.918	0.044
Var%	8.39	—	8.05	—	8.74	—
*p*‐value	< 0.0001****	—	0.0062**	—	0.0011**	—
Placebo						
T0	0.846	0.050	0.876	0.041	0.817	0.041
T30 days	0.873	0.047	0.894	0.044	0.853	0.042
Var%	3.37	—	2.26	—	4.47	—
*p*‐value	0.0306*	—	0.3404	—	0.0220*	—

*Note:* Statistically significant as *p*‐value < 0.05.

* *p* ≤ 0.05 low statistically significant.

** *p* ≤ 0.01 statistically significant.

**** *p* ≤ 0.0001 extremely statistically significant.

The difference at T0 between product and placebo is not significantly different (*p* = 0.5824), showing that the test was conducted correctly. The investigated parameter decreases by −5.76% after 30 days of product application (statistically significant). The observed reduction in *R*_0_, intended as skin firmness, which is likely correlated with skin barrier parameters, such as hydration and protein content, is consistent across the two subgroups with respective results of −5.18% and −6.33% after 30 days of product application. Additionally, for the older group, there is a contrary effect exerted by the placebo (3.04%), resulting in a net effect of −9.37% (*p* = 0.0127*).

The difference at T0 between product and placebo is not significantly different (*p* = 0.1227), showing that the test was conducted correctly. The investigated parameter globally increases by 8.39% after 30 days of product application (statistically significant). Even with the age subgroup division, the result remains consistent, with an increase in *R*_2_, representing elasticity, of 8.05% and 8.74% respectively for Yg and Og. It should be noted that for the younger group, the increase is detected only for the product, while for the older group, the placebo also shows an effect, thus determining a final net effect of 13.21% (*p* = 0.0082**).

##### 3.2.2.2 Skin Roughness

The skin surface roughness was defined by the measurement of the Maximum height (mm) of skin, and data are reported in Table 6 as mean ± standard deviation of product and placebo at different times for the entire panel as well as the age‐specific subgroups.

**TABLE 6:** Skin roughness results on product and placebo.

	Total		Yg		Og	
	Mean	SD	Mean	SD	Mean	SD
Product						
T0	0.230	0.040	0.241	0.040	0.219	0.038
T30 days	0.197	0.030	0.198	0.029	0.197	0.032
Var%	−13.63	—	−17.45	—	−9.81	—
*p*‐value	< 0.0001****	—	0.0002***	—	0.0067**	—
Placebo						
T0	0.226	0.048	0.239	0.052	0.213	0.043
T30 days	0.230	0.047	0.236	0.050	0.225	0.046
Var%	2.20	—	−1.37	—	5.78	—
*p*‐value	0.2901	—	0.4589	—	0.0458*	—

*Note:* Statistically significant as *p*‐value < 0.05.

* *p* ≤ 0.05 low statistically significant.

** *p* ≤ 0.01 statistically significant.

*** *p* ≤ 0.001 highly statistically significant.

**** *p* ≤ 0.0001 extremely statistically significant.

**FIGURE 2:**
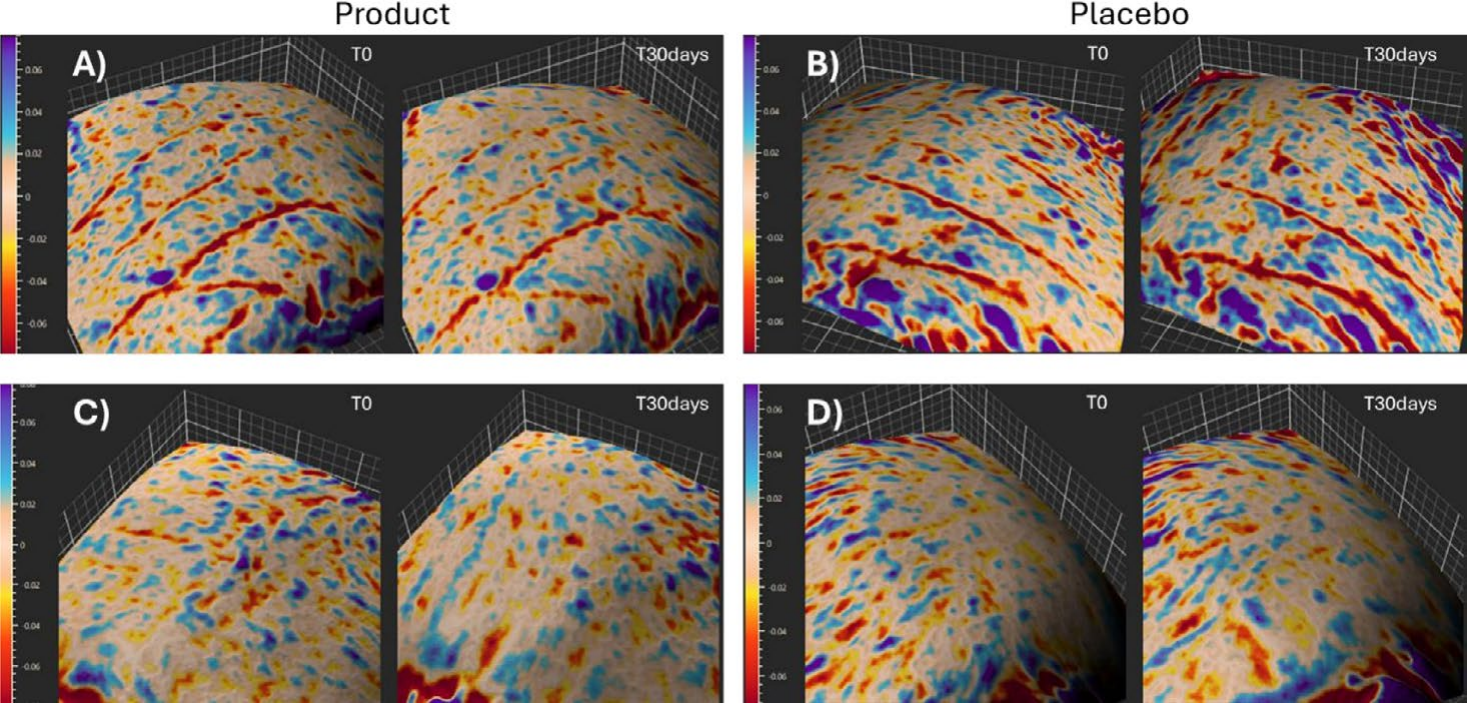
Images of skin roughness (Antera). (A) shows the before/after product treatment for an Og subject (*n*°17); (B) shows the corresponding before/after placebo treatment. (C) shows the before/after product treatment for an Yg subject (*n*°5); (D) shows the corresponding before/after placebo treatment.

The difference at T0 between product and placebo is not significantly different (*p* = 0.4883), showing that the test was conducted correctly. The investigated parameter decreases by −13.63% after 30 days of product application (statistically significant). Even with the age subgroup division, the result remains consistent, with a decrease of skin roughness, of 17.45% and 9.81% respectively for Yg and Og. It should be noted that for the older group, the placebo shows a detrimental effect, thus determining a final net effect of 15.59%. Figure 2 shows images of skin roughness elaborated through Antera software.

**FIGURE 3:**
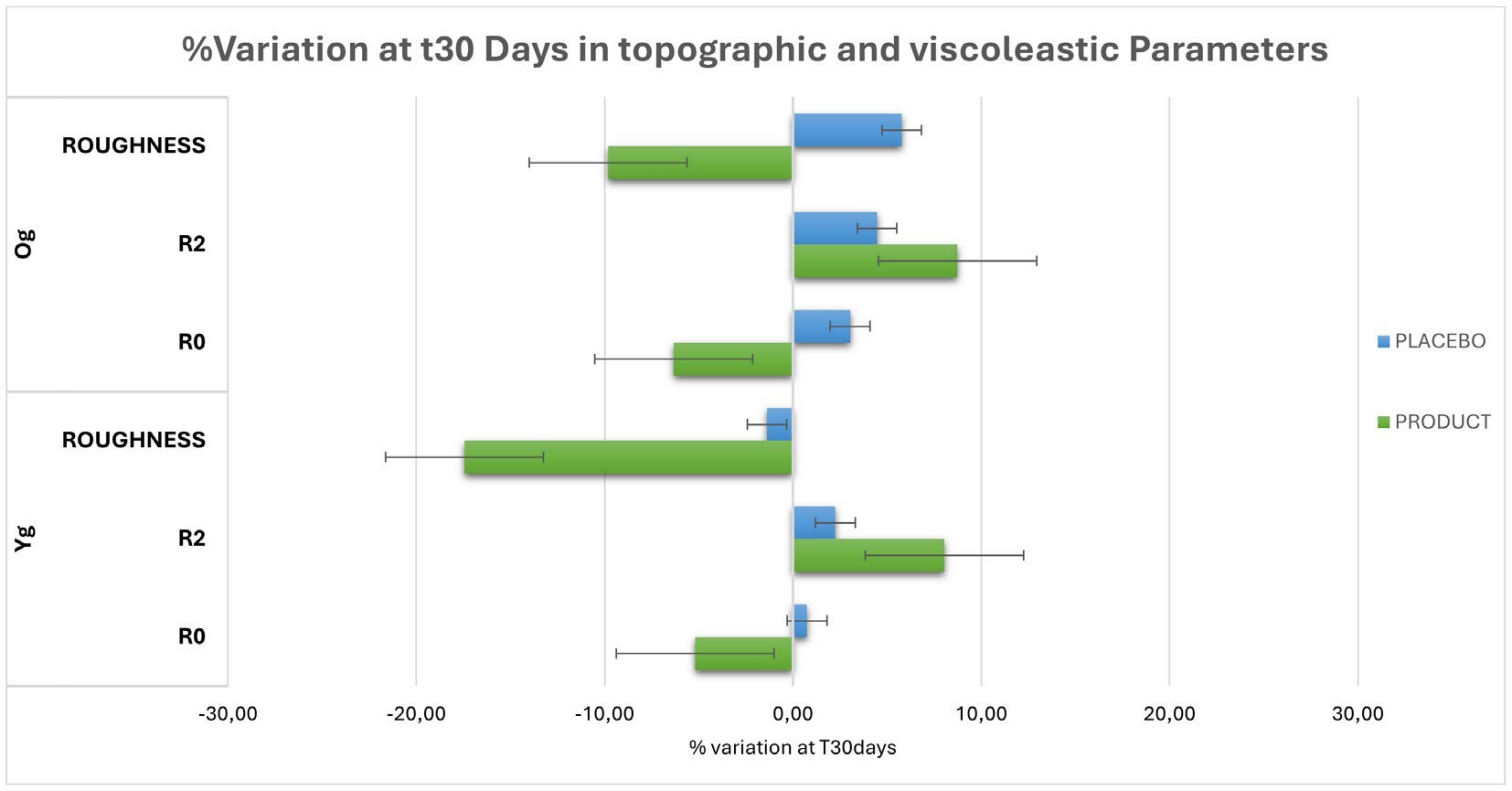
Summary of topographic and viscoelastic variations between the two age groups (Yg; Og). The image illustrates differences in roughness, *R*_0_, and *R*_2_, highlighting the outcomes across different age demographics after 30 days of product application.

Figure 3 report the summary of the results obtained from topographic and viscoelastic analyses.

### 3.3 Skin Microbiota Results

For the evaluation of skin microbiota and how it changes during treatments it is important to consider the main bacteria present starting from phylum level to species level. For this study, an additional division was made to compare how treatments change the microbial diversity based on female age. The data will be reported in Table 7 in order of groups from youngest to oldest.

**TABLE 7:** % distribution of main bacteria according to age.

	Yg T0 (%)	Og T0 (%)
Phylum		
*Actinobacteria*	16.93	22.97
*Bacteroidota*	1.63	2.50
*Cyanobacteria*	0.58	1.12
*Firmicutes*	10.21	11.93
*Proteobacteria*	69.67	59.72
Class		
*Actinobacteria*	16.78	22.73
*Bacteroidia*	1.63	2.50
*Bacilli*	8.27	8.28
*Alphaproteobacteria*	3.51	3.63
*Gammaproteobacteria*	66.12	56.07
Order		
*Propionibacteriales*	9.38	5.28
*Corynebacteriales*	3.83	8.60
*Micrococcales*	2.87	7.67
*Bacteroidales*	1.19	2.03
*Lactobacillales*	4.32	4.38
*Staphylococcales*	3.66	3.33
*Pseudomonadales*	34.58	42.43
*Burkolderiales*	30.43	12.72
Family		
*Corynebacteriaceae*	3.70	8.42
*Microbacteriaceae*	1.61	1.82
*Propionibacteriaceae*	9.33	5.23
*Streptococcaceae*	3.19	2.80
*Gemellaceae*	0.34	0.10
*Burkholderiaceae*	4.19	5.02
*Neisseriaceae*	25.69	7.22
*Pseudomonadaceae*	32.83	41.02
Genus		
*Corynebacterium*	1.83	6.90
*Lawsonella*	1.86	1.47
*Rhodococcus*	1.09	1.22
*Cutibacterium*	9.33	5.22
*Streptococcus*	3.19	2.80
*Staphylococcus*	3.32	3.22
*Burkholderia‐Caballeronia‐ Paraburkholderia*	3.62	4.38
*Pseudomonas*	32.83	41.02
Species		
*Cutibacterium acnes*	8.90	5.02
*Streptococcous oralis*	2.57	1.63
*Staphylococcus caprae*	2.43	2.67
*Burkholderia cenocepacia*	2.14	2.62
*Pseudomonas koreensis*	27.78	34.45
*Pseudomonas psychrotolerans*	1.29	1.70

As an initial analysis, it is crucial to understand the composition of skin microbiota at time 0 in the two groups characterized by different age ranges. The first group, referred to as “young,” includes individuals under 50 years of age, while the second group, referred to as “old,” includes individuals over 50 years of age. What emerges is a different percentage of *Cutibacterium acnes*, which is higher in the young group at 8.90%, compared to 5.02% in the older group. This might indicate healthier skin, as *Cutibacterium acnes* is one of the most prevalent commensal bacteria on our skin. Conversely, a microorganism present in high percentages in both groups is *Pseudomonas koreensis* , which is not a commensal bacterium but, as reported in the literature, proliferates when the skin microbiota is altered. It is characteristic of both dry skin and rosacea. The presence of this bacterium, accounting for 27.78% in the young group and 34.45% in the older group, supports the selection of the panel for this study, consisting of subjects distinguished by age but characterized by dry skin, as confirmed at the microbial level.

Subsequently, after the application of both the placebo and the product, skin flora analyses were conducted again. To better understand the behavior of skin microbiota following treatment, it is crucial to focus on species distribution. To better understand the role of placebo and product, percentage variation from t0 is reported for species level in Table 8.

**TABLE 8:** Percentage variation of species level based on placebo and product is reported.

Species	Placebo		Product	
	Yg T30 (%)	Og T30 (%)	Yg T30 (%)	Og T30 (%)
*Cutibacterium acnes*	9.90	47.83	23.47	50.50
*Streptococcus oralis*	38.07	−127.91	−21.21	−39.80
*Staphylococcus caprae*	2.67	−41.59	26.48	−30.00
*Burkholderia cenocepacia*	−3.21	−26.61	−5.70	−17.20
*Pseudomonas koreensis*	−31.86	−15.99	−32.16	−27.67
*Pseudomonas psychrotolerans*	−364.00	−175.68	−65.52	−74.51

#### 3.3.1 Microbial Analyses

##### 3.3.1.1 Good Coverage and Alpha Diversity

**FIGURE 4:**
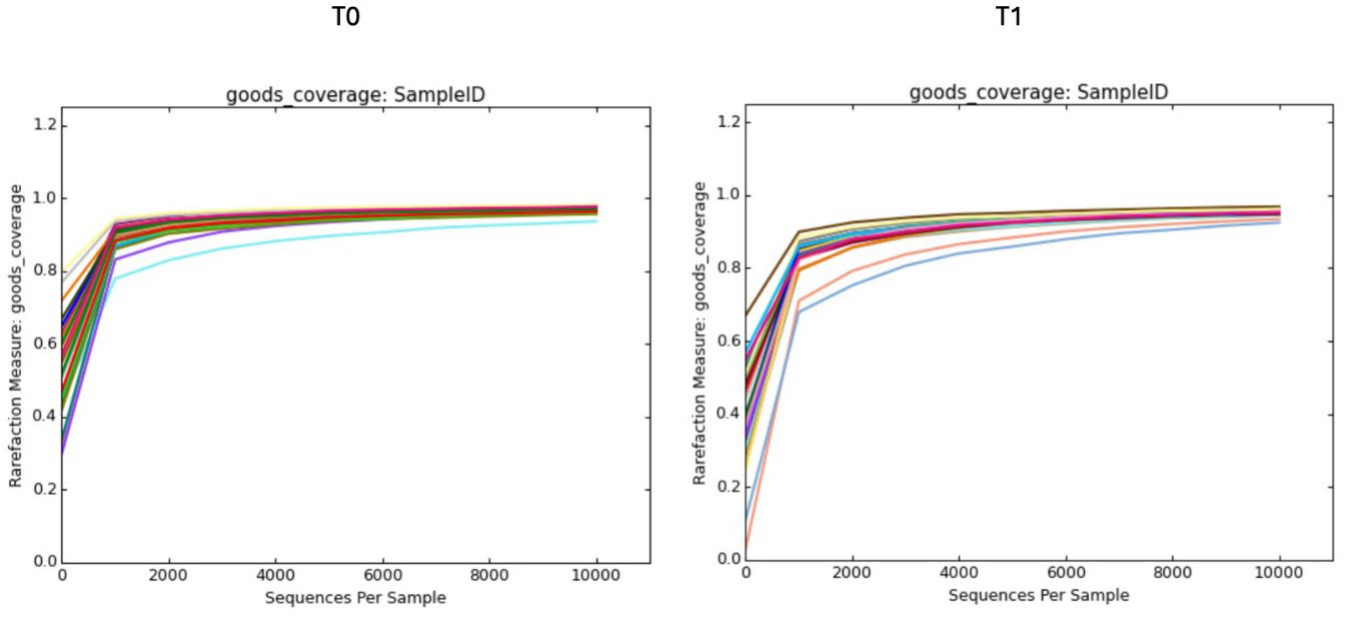
16S rRNA V1–V2 high‐throughput sequencing libraries of the panel selected. Good's coverage rarefaction curves based on sequences per sample indicate that the sequencing method was efficient with a value > 0.960 for all samples for 10 000 sequences. Left T0; right T30 days.

Figure 4 reports the coverage of the sample by distinguishing T0 and T30days. The coverage was efficient and good: in fact, the value ranges from 0.960 to 0.980.

**FIGURE 5:**
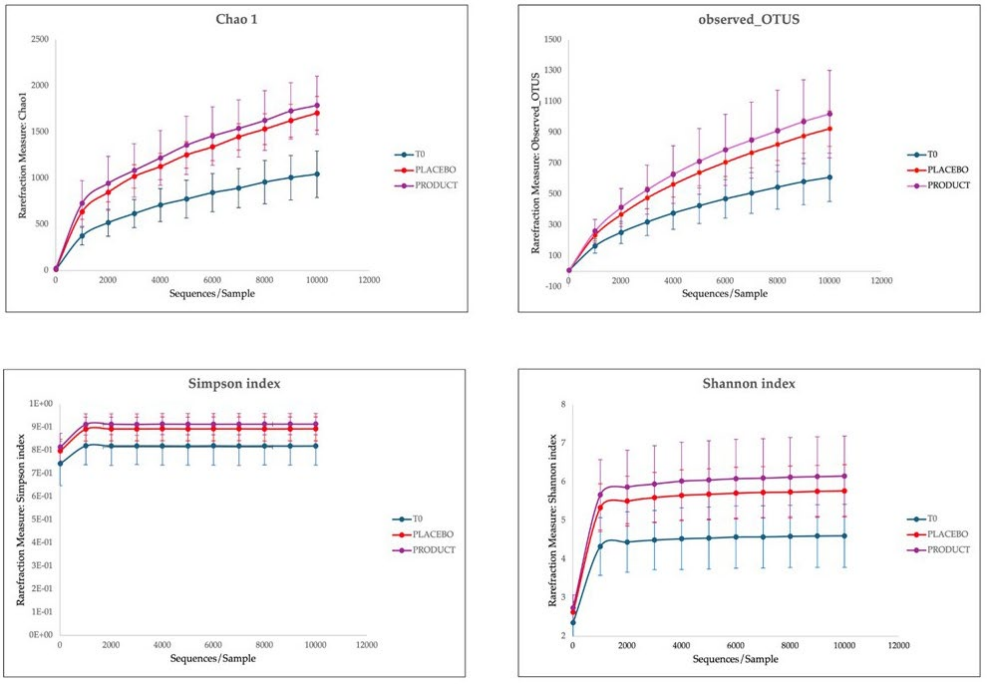
The most important indices for microbiota are reported. For all indices, there is evidence that with the application of both product and placebo, there is an increase in the richness and diversity of microbial flora. For observed OTUs, statistical *F* 74.90 > critical *F* 2.85; for Chao 1 index statistical *F* 123.43 > critical *F* 2.86; Simpson index statistical *F* is 8.50 > critical *F* 2.85; and in the end Shannon index statistical *F* 29.48 > critical *F* 2.86.

The rarefaction curve, which compares the number of different OTUs observed at different sequencing depths provides evidence that the sequencing method sufficiently detected the majority of OTUs in each sample (Figure 5). As mentioned before, “Observed OTUs” refers to the number of Operational Taxonomic Units (OTUs) that have been identified or observed in samples.

Rarefactions were performed to a depth of 1000 reads. At the maximum subsampling depth of 1000 reads, the mean observed OTUS richness was 609.31 ± 156.75 at t0, 924.51 ± 114.97 for placebo and 1019.50 ± 283.62. Rarefaction curves for richness in all group samples plateau at the maximum depth indicating an adequate sampling procedure.

Chao1 index is applied to estimate the true diversity of microbial species in each sample and in this context in each group.

Figure 5 reports rarefaction measures of the Chao 1 index compared to the sequences per sample. At the 10 000 depth sequences the mean of each group is 1043.87 ± 252.02 for T0, 1704.94 ± 182.08 for placebo and 1790.10 ± 315.51 for product. Moreover, we can affirm that the diversity of species improves with the application of both products, underlining that they help to increase the balance of skin microbiota (Figure 5).

The alpha diversity was also analyzed by measuring the Shannon and Simpson indices. In the Shannon index, the value increases with the application of the product: indeed, the value is 4.605 ± 0.82 for t0, 5.77 ± 0.67 for placebo and 6.157 ± 1.04 for product. For the Simpson index, at t0 the values are 0.819 ± 0.082; 0.893 ± 0.052 for placebo and 0.913 ± 0.046 for product. Observing the graph, it is evident that for all indices, there is an increase in heterogeneity following the application of the product and the placebo. Indeed, for observed OTUs, Chao 1, Simpson, Shannon indices, the statistical *F* is greater than the critical *F*, with a *p*‐value < 0.05.

## 4 Discussion

Dry skin is commonly characterized by a compromised skin barrier, with reduced corneocyte cohesion leading to increased TEWL, and is often accompanied by microbiota imbalance. The skin microbiota is a dynamic and site‐specific microbial ecosystem that colonizes both the surface and deeper layers of the epidermis [22]. It plays a fundamental role in maintaining immune homeostasis, protecting against pathogens, and modulating inflammatory responses—similarly to the gut microbiome. However, due to its direct exposure to the environment, the cutaneous microbiota is highly variable and influenced both by intrinsic and extrinsic factors. In the context of dry skin, microbial imbalance is typically marked by a dysbiosis that may exacerbate barrier impairment and contribute to chronic inflammation, thereby reinforcing the vicious cycle of dryness [23]. Therefore, ingredients such as prebiotics, probiotics, and postbiotics have gained attention for their cosmetic potential in restoring microbial homeostasis and supporting skin function.

This study evaluated the effects of a topical formulation ‐Nourish 3‐ Biotic Rich Serum‐ on the skin microbiota and cutaneous outcomes in subjects with dry skin. Through a combined approach that included alpha diversity analysis, skin parameters, and stratification by age, the preliminary results demonstrate that the application of the serum containing prebiotics, probiotics, and postbiotics led to improvements in biophysical parameters of the skin—across different ages as well as changes in microbial flora compared to a placebo.

The observed increase in skin hydration (16.94% after 30 days) is indicative of a reinforced skin barrier, which is a crucial aspect in the management of dry skin conditions. Additionally, the decrease in stratum corneum protein content (−14.57% after 30 days) suggests a normalization of the desquamation process, as well as skin cohesion and a cellular renewal action. Changes in skin firmness and elasticity (*R*_0_: −5.76%; *R*_2_: 8.39% after 30 days) accompanied by reductions in skin roughness (−13.63% after 30 days), also suggest action on the mechanical and topographical features of the skin. These findings are in line with studies reporting that topical application of probiotics or prebiotics can improve skin barrier properties and skin roughness [24]. Another key result of this study is the observed increase in microbial diversity, considering that microbiota heterogeneity is often associated with greater skin health [25]. Namely, for microbial analysis, subjects were stratified by age (< or > 50 years) to verify the possible different behavior and features of skin microbiota at t0 and then with the use of the product. In fact, the skin microbiome can change with age, and its imbalance can contribute to skin aging [26]. At baseline, in agreement with other studies, Proteobacteria, Firmicutes, and Actinobacteria were the most represented phyla across both age groups [27]. This confirms that facial skin is typically colonized by a core group of microbial taxa whose distribution and abundance can deeply vary [28]. Notably, *Cutibacterium acnes* isone of the primary commensal bacteria of the skin thatmetabolizes fatty acids possessing antimicrobial properties and aids in maintaining the acidic pH of the skin. Moreover, it produces bacteriocins, which inhibit the growth of yeasts, molds, and certain Gram‐negative pathogens. At baseline, the younger group showed higher levels of *Cutibacterium acnes* (8.90%) compared to the older group (5.02%), suggesting better skin condition and microbial balance in younger participants since it is a key player in skin homeostasis [29]. Conversely, *Pseudomonas koreensis* , often associated with dry and dysbiotic skin, was present at elevated levels in both age groups (27.78% in < 50 and 34.45% in > 50), indicating a shared microbiota alteration across the panel. After 30 days of treatment, both the placebo and active serum increased alpha diversity, as verified with the Simpson, Shannon and Chao1 index helping in the restoration of the skin microbiota. However, only Nourish 3‐Biotic Rich Serum promoted significant improvements in key microbial populations. Specifically, it can increase the quantity of *Cutibacterium acnes* with a percentage of 23.47% in the young group and 50.50% in the old group. At the same time, *Pseudomonas koreensis* , usually associated with dry and rosacea skin, starting from 27.78% in the first group (< 50 y.o) and 34.45% in the second group (> 50 y.o.) at the baseline was reduced by −32.16% and −27.67%, respectively. This could be due probably to a lack of sebum characteristic of dry skin. These shifts reflect a dual effect: restoration of beneficial commensals and suppression of opportunistic species. Such microbial modulation is consistent with current literature, which shows that biotic actives—particularly prebiotics and postbiotics—can promote moisture retention, modulate inflammation and strengthen the skin barrier by reshaping the skin microbiome [25, 30]. Interestingly, the alpha diversity findings from this study align with emerging evidence suggesting that age‐related trends in microbiota diversity are not linear or universal. Some studies report increased diversity in older subjects, others in younger ones, while intermediate age groups often exhibit distinct microbial signatures. These discrepancies may be explained by external factors such as geography, urbanization, or skin condition, supporting the notion that age alone is not the primary driver of microbiome changes [28]. Altogether, these data support the hypothesis that customized cosmetic treatments may condition the skin microbiota toward a more eubiotic state, regardless of the user's age or initial microbial profile. This is further reinforced by the absence of significant differences in post‐treatment outcomes between age groups. Similar results were reported by Ju Hee Han et al. [24], who demonstrated that prebiotic‐based interventions increased microbial richness and rebalanced dysbiotic skin, particularly in cases of dryness and sensitivity. These findings reinforce the potential of microbiota‐targeted strategies as a supportive approach in the management of dry skin.

## 5 Conclusion

An in vivo study was performed to evaluate skin parameters and microbial data in a panel of female subjects with dry skin using Nourish 3‐ Biotic Rich Serum, containing prebiotics, probiotics, and postbiotics compared to a placebo product. The study demonstrated the improvement in hydration, elasticity, and firmness while reducing TEWL, wrinkle depth, and protein content. Moreover, it promoted greater bacterial diversity, suggesting a rebalancing effect on the microbiota. These results support the use of the cosmetic serum rich in biotic ingredients for people with dry skin and microbiota imbalance, regardless of age. Future studies could explore larger populations to validate these preliminary findings that have been observed.

## Author Contributions

Conceptualization, G.G. and C.D.; methodology, P.P.; formal analysis, G.D. and G.C.; investigation, S.B. and P.P.; writing – original draft preparation, P.P. and G.C.; writing – review and editing, P.P., S.B., G.G., F.L.B., and C.D.; supervision, C.D.; project administration, C.D. All authors have read and agreed to the published version of the manuscript.

## Ethics Statement

The authors have nothing to report.

## Consent

Informed consent was obtained from all subjects involved in the study.

## Conflicts of Interest

Camilla D'Antonio is co‐founder, stakeholder and scientific director of Medspa S.r.l. Giulia Gentili, Fabrizia Lo Bosco and Serena Belmonte are employees of said company. Medspa S.r.l. is an Italian company owner of the Nourish 3‐Biotic Rich Serum.

## Data Availability

The data that support the findings of this study are available on request from the corresponding author. The data are not publicly available due to privacy or ethical restrictions.
